# Increased *Fusobacterium* tumoural abundance affects immunogenicity in mucinous colorectal cancer and may be associated with improved clinical outcome

**DOI:** 10.1007/s00109-023-02324-5

**Published:** 2023-05-12

**Authors:** William P. Duggan, Manuela Salvucci, Batuhan Kisakol, Andreas U. Lindner, Ian S. Reynolds, Heiko Dussmann, Joanna Fay, Tony O’Grady, Daniel B. Longley, Fiona Ginty, Elizabeth Mc Donough, Daniel J. Slade, John P. Burke, Jochen H. M. Prehn

**Affiliations:** 1 Department of Colorectal Surgery, Beaumont Hospital, Dublin 9, Ireland; 2 Department of Physiology and Medical Physicsand, RCSI Centre for Systems Medicine , Royal College of Surgeons in Ireland, Dublin 2, Ireland; 3 Centre for Systems Medicine, Royal College of Surgeons in Ireland, Dublin 2, Ireland; 4 RCSI Biobank, Royal College of Surgeons in Ireland, Dublin, Ireland; 5 Centre for Cancer Research & Cell Biology, Queen’s University Belfast, Belfast, Northern Ireland UK; 6 GE Research, Niskayuna, NY 12309 USA; 7 Department of Biochemistry, Virginia Polytechnic Institute and State University, Blacksburg, VA USA

**Keywords:** Mucinous colorectal cancer, *Fusobacterium*, Microsatellite instability

## Abstract

**Abstract:**

There is currently an urgent need to identify factors predictive of immunogenicity in colorectal cancer (CRC). Mucinous CRC is a distinct histological subtype of CRC, associated with a poor response to chemotherapy. Recent evidence suggests the commensal facultative anaerobe *Fusobacterium* may be especially prevalent in mucinous CRC. The objectives of this study were to assess the association of *Fusobacterium* abundance with immune cell composition and prognosis in mucinous CRC. Our study included two independent colorectal cancer patient cohorts, The Cancer Genome Atlas (TCGA) cohort, and a cohort of rectal cancers from the Beaumont RCSI Cancer Centre (BRCC). Multiplexed immunofluorescence staining of a tumour microarray (TMA) from the BRCC cohort was undertaken using Cell DIVE technology. Our cohorts included 87 cases (13.3%) of mucinous and 565 cases (86.7%) of non-mucinous CRC. Mucinous CRC in the TCGA dataset was associated with an increased proportion of CD8 + lymphocytes (*p* = 0.018), regulatory T-cells (*p* = 0.001) and M2 macrophages (*p* = 0.001). In the BRCC cohort, mucinous RC was associated with enhanced CD8 + lymphocyte (*p* = 0.022), regulatory T-cell (*p* = 0.047), and B-cell (*p* = 0.025) counts. High *Fusobacterium* abundance was associated with an increased proportion of CD4 + lymphocytes (*p* = 0.031) and M1 macrophages (*p* = 0.006), whilst M2 macrophages (*p* = 0.043) were under-represented in this cohort. Patients with increased *Fusobacterium* relative abundance in our mucinous CRC TCGA cohort tended to have better clinical outcomes (DSS: likelihood ratio *p* = 0.04, logrank *p* = 0.052). *Fusobacterium* abundance may be associated with improved outcomes in mucinous CRC, possibly due to a modulatory effect on the host immune response.

**Key messages:**

• Increased *Fusobacterium* relative abundance was not found to be associated with microsatellite instability in mucinous CRC.

• Increased *Fusobacterium* relative abundance was associated with an M2/M1 macrophage switch, which is especially significant in mucinous CRC, where M2 macrophages are overexpressed.

• Increased *Fusobacterium* relative abundance was associated with a significant improvement in disease specific survival in mucinous CRC.

• Our findings were validated at a protein level within our own in house mucinous and non-mucinous rectal cancer cohorts.

**Supplementary Information:**

The online version contains supplementary material available at 10.1007/s00109-023-02324-5.

## Introduction

Mucinous colorectal cancer (CRC) is a histological subtype of colorectal adenocarcinoma, which accounts for approximately 5–15% of all colorectal tumours [1]. These tumours are characterised by an abundance of extracellular mucin, which constitutes more than 50% of the tumour volume [2, 3]. When compared with non-mucinous rectal cancer (RC), mucinous rectal adenocarcinoma is associated with reduced rates of pathological complete response (pCR) and tumour downstaging following neoadjuvant chemoradiotherapy. As a consequence, patients with this disease have an increased likelihood of having a positive resection margin and are associated with poorer definitive outcomes [4]. Mucinous adenocarcinoma of the colon is associated with an increased risk of metastasis, and this cohort has also been shown to be associated with resistance to oxaliplatin and irinotecan-based chemotherapy [5]. Our group have previously demonstrated that mucinous CRCs are more likely to harbour *KRAS* and *BRAF* mutations, and are more likely to demonstrate microsatellite instability (MSI), and be of the CPG island methylator phenotype as compared to non-mucinous colorectal tumours [6].

*Fusobacterium* are a genus of gram-negative facultative anaerobes, commonly encountered in gastrointestinal tract pathologies such as inflammatory bowel disease and cancer [7, 8]. The species of *Fusobacterium* most commonly associated with colorectal cancer is *Fusobacterium nucleatum (F. nucleatum)* [7]*. F. nucleatum* has been shown to be more abundant in colorectal tumour tissue compared with matched adjacent normal mucosa, which has led to the suggestion there may be a potential causative relationship [9, 10]. *F. nucleatum* promotes a pro-inflammatory state [11], and has been shown in pre-clinical studies to modulate the T-cell-mediated immune response in CRC [12]. *F. nucleatum* is also known to be more abundant in MSI-high tumours [13]. This is pertinent; given the emergence of evidence demonstrating an association between an increased abundance of various members of the gut microbiome with improved rates of responsivity to immunotherapy in cancer [14, 15].

The prognostic impact of *F. nucleatum* has been evaluated in a number of cohort studies [16]. Though some studies managed to demonstrate correlation between *F. nucleatum* abundance and poor prognosis [17, 18], this association was not observed in other studies [16, 19]. A recent publication by our group, premised on the hypothesis that the impact of *F. nucleatum/Fusobacteriales* may differ according to underlying tumour biology, demonstrated a correlation between increased *Fusobacterium* abundance and poor prognosis in mesenchymal-type tumours only [11]. A previous whole genome sequencing study, again undertaken by our group, examined 10 mucinous rectal adenocarcinomas, and found *F. nucleatum* to be significantly more abundant within mucinous tumour tissue [20]. *F. nucleatum* has previously been shown to promote MUC2, TNF-α and mucin production in colonic cells [21]. *F. nucleatum* has also been implicated in exacerbations of chronic obstructive pulmonary disease, where it has been shown to contribute towards inflammation and mucin production [22].

In light of this evidence, it was hypothesised there may be a relationship between *Fusobacterium* abundance and outcomes in mucinous CRC. The aims of this study were to compare *Fusobacterium* relative abundance at a genus rank level between mucinous and non-mucinous CRC cohorts within the Cancer Genome Atlas (TCGA) dataset, and examine whether an association exists between *Fusobacterium* abundance and immune cell composition and prognosis in mucinous CRC. We then sought to validate TCGA findings at a protein level, by hyperplexing tumour microarrays (TMAs) which included our own mucinous and non-mucinous cohort from the Beaumont RCSI Cancer Centre (BRCC) with a pan-*fusobacterium* antisera alongside an array of immune markers.

## Materials and methods

### TCGA gene expression analysis

We performed a search of TCGA to identify cases of CRC eligible for inclusion. Institutional approval was not required for these open-access data. The inclusion criteria specified stages 1 to 4; mucinous and non-mucinous colorectal adenocarcinoma. Patients of both genders and all age groups and ethnicities were eligible for inclusion in the analysis. The demographic, pathologic, and clinical data for each eligible patient were collated and harmonised from the GDC Legacy Archive and the TCGA-Clinical Data Resource publication [23]. This study focused on the impact of *Fusobacterium* in mucinous adenocarcinoma, thus we restricted the analysis to patients of the TCGA-Colorectal Adenocarcinoma (COAD)-Rectal Adenocarcinoma (READ) cohort that have both clinical information and *Fusobacterium* data (*n* = 594 of 631 candidate cases (94%)). The pathologic variables recorded included TNM stage, lymphovascular invasion (LVI), extramural vascular invasion (EMVI) and MSI status. Patients were also categorised according to consensus molecular subtype [24]. Regarding survival, three end-points were considered; disease-free survival (DFS), disease-specific survival (DSS), and overall survival (OS).

*Fusobacterium* composition was derived from RNASeq experiments as previously described by Salvucci et al. [11]. Briefly, composition was computationally inferred using PathSeq from RNA experiments by aligning reads not mapped to the host to microbial taxonomic references [25]. We reported Fusobacterium relative abundance (in %) at the genus and species taxonomic ranks. Sub-species/strains reported by PathSeq were remapped to their parent species following sequence blasting, as described in Salvucci et al. [11]. Level 4 transcriptomic data were retrieved from TCGA pancancer release and subset to include only measurements from primary tumours of patients diagnosed with COAD and READ. Cell type composition was computed using the quanTIseq package, as previously described [11].

### Immunofluorescence staining of tumour microarrays

Formalin-fixed, paraffin-embedded primary tumour tissue sections were obtained from patients with stage I-III rectal cancer following tumour resection at the BRCC. Tissue was provided from the Beaumont Hospital Colorectal Biobank with written consent provided by all patients. Institutional ethical approval was granted by the Beaumont Ethics (Medical Research) Committee (Reference 21/98). Mucinous tumours were defined by a consultant histopathologist as those with greater than 50% of the tumour composed of mucin. To construct the TMA 1 mm punches were taken from different regions within the centre of the tumour. Multiplexed immunofluorescence staining of the tumour micro-array with relevant immune markers (CD3, CD4, CD8, CD20 and forkhead box P3 (FOXP3)) were performed using Cell DIVE™ technology (Leica Microsystems, Issaquah, USA). This involves multiple rounds of antibody staining performed on the same tissue section with mild dye oxidation between successive rounds of staining and imaging [26]. Epithelial cells were segmented using stains against DAPI, and antibodies for pan-cytokeratin (CK-26), ribosomal protein S6 (S6) and Na^+^K^+^ATPase and stromal cells were segmented using DAPI. Antibodies were acquired commercially and underwent a multi-step process of validation and conjugation (as previously described by Gerdes et al. [26]). Detailed description of the image analysis workflow was published in a larger analysis of 373 tumour cores by our research group [27]. To summarise, immune cells were classified according to cell-level expression and were quantified at tissue core (tumour and stroma) patient level [27]. Immune cell composition in the tumour cores varied significantly, with some cores showing predominantly cancerous/epithelial cells in the absence of immune cell infiltration, and others showing very high levels (up to 55%) of immune cells. A bootstrap analysis using randomly sampled pairings; found cell type composition in cores from the same patient, to be more similar to each other compared to random pairings, suggesting that cell type composition was a biological feature of individual tumours.

### Pan-fusobacterium outer membrane antisera

A pan-fusobacterium outer membrane antisera was produced and made available by Professor Slade’s group (Department of Biochemistry, Virginia Polytechnic Institute and State University, Blacksburg, VA 24,061, USA) [28]. Staining of the TMAs with the pan-fusobacterium membrane antisera was conducted separately using the GeoMx imaging platform (Nanostring, Seattle, WA, USA). Separate tissue sections from the corresponding TMAs underwent standard deparaffinisation, ahead of antigen target retrieval using 1X Tris EDTA (PH 9.0) (Abcam). Microarrays were then fixed using 10% neutral buffered formalin. Slides were next blocked with Buffer W (Nanostring) for 30 min at 37 °C. This was followed by the addition of 1:300 dilution of the antisera for 1 h at 37 °C. Slides were next washed twice for 2 min in 2 × SSC wash buffer and incubated for 1 h with Alexa Fluor 594 goat anti-rabbit antibody (Abcam) diluted in buffer W (Nanostring). After 3 further washes with 2 × SSC, TMAs were finally incubated for 1 h with Syto 13 conjugated to Alexa Flour 488 (Nuclear stain) (Nanostring) and pancytokeratin conjugated to Alexa Flour 532 (Nanostring). Exposure time was set to 300 ms for the 594 and 532 channel and 100 ms for the 488 channel. Images were analysed using FIJI imaging software [29]. Images were thresholded and area of staining intensity quantified [30]. To verify the validity of the antisera, sections from 8 tumours which had previously undergone whole genome sequencing, were stained with the antisera. *Fusobacterium* burden was quantified and levels were compared with *Fusobacterium* relative abundance as interpreted previously from existing sequencing data [20].

### Statistical analysis

**Fig. 1:**
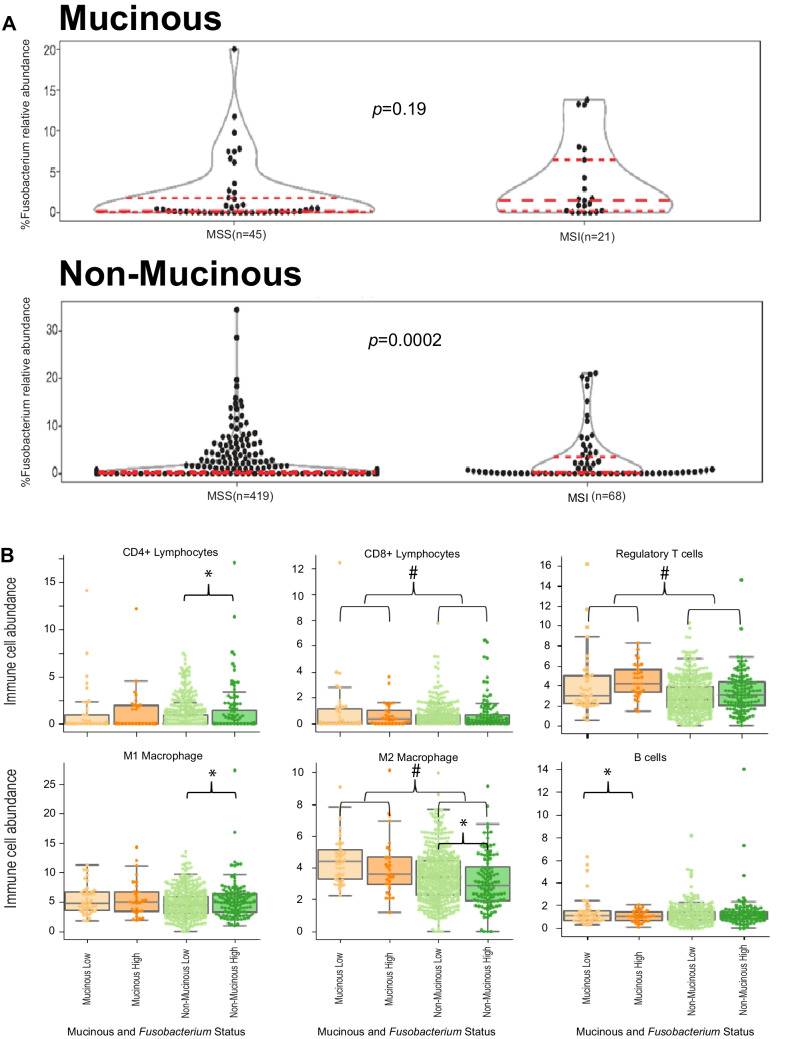
*Fusobacterium* relative abundance was found to impact immunogenicity, prognosis and MSI status in mucinous and non-mucinous CRC in the TCGA-COAD-READ cohort. **A** Violin plots depicting *Fusobacterium* relative abundance within the TCGA-COAD-READ cohort according to mucinous status and MSI status. Median, lower (25th) and upper (75th) percentiles are indicated by dashed lines. Statistical significance was evaluated using Kruskal–Wallis tests and *p*-values are reported. **B** Box and whisker plots depicting specific immune cell counts; according to mucinous status and *Fusobacterium* relative abundance (high and low) within the TCGA-COAD-READ cohort. Statistical significance was evaluated using Kruskal–Wallis tests. * indicates a statistically significant difference between *Fusobacterium* high and *Fusobacterium* low cohorts. # indicates a statistically significant difference between mucinous and non-mucinous cohorts

For the statistical analysis reported in Fig. 1A, we used *Fusobacterium* relative abundance as a continuous variable. For all other analyses, we grouped patients into high versus low *Fusobacterium* relative abundance. We defined high versus low Fusobacterium relative abundance using the 75th percentile as a cut-off. This cut-off was determined from the data and is in agreement with cut-offs used previously in the literature [11].

**Fig. 2:**
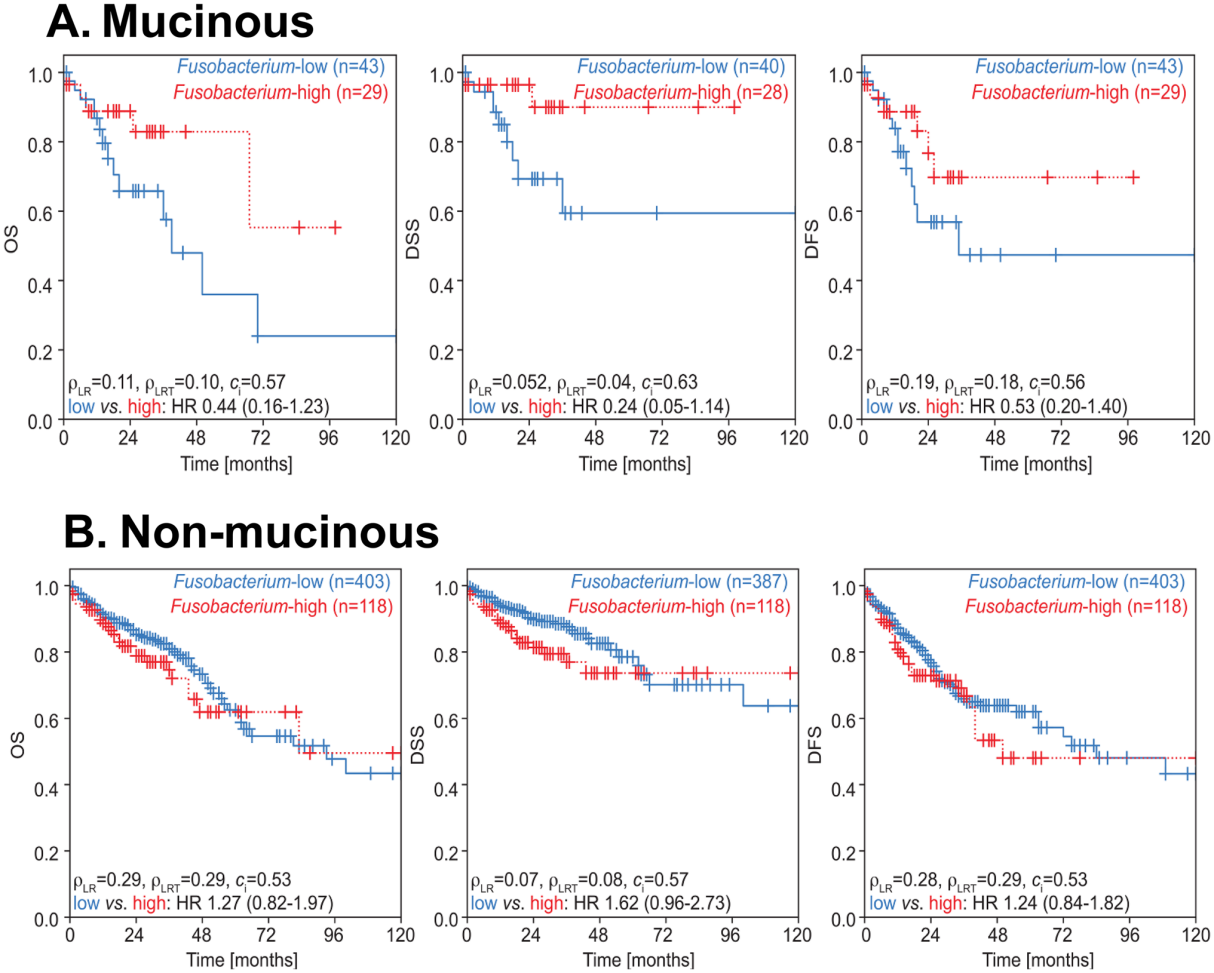
Kaplan Meier Curves depicting survival differences between patients grouped by mucinous status and by *Fusobacterium* relative abundance. Differences in survival outcomes were assessed by logrank tests (p_LR) and univariate Cox proportional hazard models (hazard ratio, 95% CI and likelihood ratio test p-value (p_LRT) and concordance index (c_i))

Continuous variables were reported as medians with interquartile ranges (IQRs). Group comparisons of continuous variables were determined by Kruskall-Wallis test. Categorical variables were reported as numbers with percentages, group comparisons of categorical variables were determined by Fisher’s exact tests. Differences between *Fusobacterium* relative abundance by group were determined using a non-negative binomial test (function GLM.NB from the MASS R package) (Fig. 1A). Association between expression of cell types with mucinous status and Fusobacterium relative abundance was assessed by fitting a linear regression model with cell type abundance (continuous, in %) as the response variable and the mucinous status (mucinous vs. non-mucinous), Fusobacterium relative abundance (binary, low vs. high) and the interaction between mucinous status and Fusobacterium relative abundance as predictor terms. We report effect sizes, 95% confidence intervals and likelihood ratio p-values, (Table 2). To avoid overfitting in the BRCC cohort, we conducted variance analyses, and reported *p*-values only (Table 4). Differences in survival according to *Fusobacterium* relative abundance were assessed by logrank tests (p_LR_) and univariate Cox proportional hazard models for which we reported hazard ratio, 95% CI and likelihood ratio test *p*-value (p_LRT_) and concordance index (c_i_), (Fig. 2). Cox regression models were fitted on relative abundance of species from the Fusobacterium genus (binary, low vs. high) by clinical endpoint. The low subgroup was used as reference when reporting the hazard ratios (HRs) estimated from the Cox regression models. Univariate Cox regression models were fitted when evaluating the association between species subgroups in the whole unselected patient population. Cox regression models with an interaction term between species subgroup (binary, low vs. high) and mucinous status (mucinous vs. non-mucinous) were fitted to evaluate differential effect of species relative abundance on clinical outcome by mucinous status (Sup. Fig. 1). A *p*-value of < 0.05 was defined as the cut off for statistical significance unless otherwise stated. Data pre-processing and analysis was performed in Python (version 3.8.10, Python Software Foundation, Wilmington, DE, USA), unless otherwise stated.

## Results

### Increased *Fusobacterium* relative abundance was not associated with MSI status in mucinous CRC in the TCGA dataset

From the TCGA dataset, we included 72 cases (12%) of mucinous and 522 cases (88%) of non-mucinous CRC. The clinical and pathologic characteristics of the included patients are summarised in Table 1. We found mucinous tumours were more likely to be proximal (*p* < 0.001) and had a higher incidence of MSI compared to the non-mucinous CRC group (*p* < 0.001). The two cohorts also demonstrated significant differences when categorised by consensus molecular subtype (*p* < 0.001). Mucinous tumours were over-represented in CMS1 (immune) (26.4% vs 11.7%) and CMS4 (mesenchymal) (31.9% vs 23%) categories and under-represented in the CMS2 (canonical) group. Following an investigation into whether the relative abundance (RA) of *Fusobacterium* at the genus taxonomic rank differed between mucinous and non-mucinous CRC, we found a trend, albeit not statistically significant, whereby patients with mucinous CRC trended to have higher *Fusobacterium* relative abundance compared to patients with non-mucinous CRC (*p* = 0.07).

**Table 1:** Clinical characteristics of the patients of the TCGA-COAD-READ cohort. CRC, colorectal cancer; AJCC, American Joint Committee on Cancer; CMS, consensus molecular subtypes; IQR, inter-quartile range. Categorical data reported as *n* (%). Continuous data reported as median (IQR)

		**Mucinous CRC** **(*n* = 72)**	**Non-mucinous CRC** **(*n* = 522)**	***p*-value**
**Age**		67 (52–77.5)	68 (58–76)	0.611
**Sex**	Male	37 (51.4%)	241 (46.2%)	0.48
	Female	35 (48.6%)	281 (53.8%)	
**Tumor location (*n* = 579)**^**a**^	Colon	58 (81.7%)	366 (72%)	0.115
	Rectum	13 (18.3%)	142 (28%)	
**Stage (*n* = 574)**^**a**^	AJCC 1	10 (14.1%)	90 (17.9%)	0.571
	AJCC 2	28 (39.4%)	189 (37.6%)	
	AJCC 3	25 (35.2%)	147 (29.2)	
	AJCC 4	8 (11.3%)	77 (15.3%)	
**T Stage (*n* = 593)**^**a**^	T1	2 (2.8%)	18 (3.5%)	0.528
	T2	9 (12.5%)	90 (17.3%)	
	T3	50 (69.4%)	358 (68.7%)	
	T4	11 (15.3%)	55 (10.6%)	
**N Stage (*n* = 591)**^**a**^	N0	39 (54.2%)	297 (57.2%)	0.528
	N1	16 (22.2%)	128 (24.7%)	
	N2	17 (23.6%)	94 (18.1%)	
**M Stage (*n* = 525)**^**a**^	M0	51 (86.4%)	389 (83.5%)	0.693
	M1	8 (13.6%)	77 (16.5%)	
**Resection margin (*n* = 438)**^**a**^	R0	51 (96.2%)	381 (99%)	0.157
	R1/R2	2 (3.8%)	4 (1%)	
**Lymphovascular invasion (*n* = 535)**^**a**^	Yes	22 (33.8%)	197 (41.9%)	0.269
	No	43 (66.2%)	273 (58.1%)	
**Perineural invasion (*n* = 230)**^**a**^	Yes	6 (22.2%)	54 (26.6%)	0.800
	No	21 (77.8%)	149 (73.4%)	
**Vascular invasion (*n* = 514)**^**a**^	Yes	12 (20%)	115 (25.3%)	0.459
	No	48 (80%)	339 (74.7%)	
**Microsatellite status (*n* = 553)**^**a**^	MSI	21 (31.8%)	68 (14%)	** < 0.001**
	MSS	45 (68.2%)	419 (86%)	
**CMS (*n* = 536)**^**a**^	CMS1	19 (26.4%)	61 (11.7%)	** < 0.001**
	CMS2	4 (5.6%)	234 (44.8%)	
	CMS3	20 (27.8%)	55 (10.5%)	
	CMS4	23 (31.9%)	120 (23%)	

^a^Data not available in full cohort: *n* in parentheses = number with data available

Next, we sought to investigate the relationship between *Fusobacterium* abundance and MSI status. In keeping with pre-existing evidence, when we restricted our analysis to non-mucinous CRC cases, we found MSI tumours to be strongly associated with an increased abundance of *Fusobacterium* (*p* < 0.001) (Fig. 1A). No statistically significant association was evident between *Fusobacterium* relative abundance and MSI status in the mucinous cohort (*p* = 0.19, Fig. 1A).

### Mucinous status and elevated *Fusobacterium* relative abundance were both independently found to impact composition of immune cells in the TCGA CRC cohort

Overall, mucinous tumours were associated with a significantly greater ratio of total immune cells to epithelial/stromal cells in the TCGA dataset (*p* < 0.001) (Table 2, Fig. 1B). Specifically, the mucinous cohort were associated with significantly greater proportions of CD8 + lymphocytes (*p* = 0.018), regulatory T-cells (*p* < 0.001), and M2 macrophages (*p* = 0.003) (Table 2, Fig. 1B).

**Table 2:** Immune cell expression in the TCGA cohort according to mucinous status and *fusobacterium* relative abundance as computed using the quanTIseq package

Cell Type	Level	Coefficient	Lower 95% CI	Upper 95% CI	*p*-value
**Epithelium/stroma**	Mucinous status(mucinous) *Fusobacterium* (high) Mucinous status(mucinous): *Fusobacterium*(high)	− 3.70 − 1.30 1.8	− 5.80 − 2.60 − 1.60	1.70 0.06 5.10	** < 0.001** 0.061 0.297
**CD4 + lymphocytes**	Mucinous status(mucinous) *Fusobacterium* (high) Mucinous status(mucinous): *Fusobacterium*(high)	0.40 0.42 − 0.30	− 0.19 0.04 − 1.20	0.98 0.79 0.65	0.182 **0.032** 0.538
**CD8 + lymphocytes**	Mucinous status(mucinous) *Fusobacterium* (high) Mucinous status(mucinous): *Fusobacterium* (high)	0.42 0.14 − 0.36	0.07 − 0.08 − 0.92	0.76 0.37 0.20	**0.018** 0.207 0.204
**T Regs**	Mucinous status(mucinous) *Fusobacterium* (high) Mucinous status(mucinous): *Fusobacterium* (high)	1.1 0.39 0.12	0.46 < − 0.01 − 0.87	1.70 0.78 1.10	** < 0.001** 0.053 0.807
**Dendritic cells**	Mucinous status(mucinous) *Fusobacterium* (high) Mucinous status(mucinous): *Fusobacterium* (high)	− 0.02 0.03 − 0.04	− 0.15 − 0.05 − 0.25	0.11 0.12 0.17	0.744 0.458 0.713
**B cells**	Mucinous status(mucinous) *Fusobacterium* (high) Mucinous status(mucinous): *Fusobacterium* (high)	0.28 0.14 − 0.54	− 0.03 − 0.06 − 1.00	0.59 0.35 − 0.04	0.078 0.159 **0.035**
**M1 macrophage**	Mucinous status(mucinous) *Fusobacterium* (high) Mucinous status(mucinous): *Fusobacterium* (high)	0.63 0.73 − 0.39	− 0.18 0.2 − 1.7	1.40 1.30 0.93	0.129 **0.007** 0.565
**M2 macrophage**	Mucinous status(mucinous) *Fusobacterium* (high) Mucinous status(mucinous): *Fusobacterium* (high)	0.96 − 0.35 < − 0.01	0.44 − 0.69 − 0.85	1.5 − 0.01 0.85	** < 0.001** **0.040** 0.990
**Natural killer cells**	Mucinous status(mucinous) *Fusobacterium* (high) Mucinous status(mucinous): *Fusobacterium* (high)	− 0.03 − 0.09 0.01	− 0.41 − 0.34 − 0.62	0.36 0.16 0.63	0.899 0.484 0.976
**Neutrophils**	Mucinous status(mucinous) *Fusobacterium* (high) Mucinous status(mucinous): *Fusobacterium* (high)	0.31 − 0.16 − 0.25	− 0.96 − 0.80 − 1.90	1.00 0.49 1.40	0.951 0.635 0.756

Tumours with high *Fusobacterium* relative abundance were found to be associated with significantly greater proportions of CD4 + lymphocytes (*p* = 0.031) and M1 macrophages (*p* = 0.006), whilst M2 macrophages *(p* = 0.043) were under-represented across this group (Table 2, Fig. 1B). Evaluation of the mucinous cohort in isolation, found a significant reduction in the proportion of B-cells (*p* = 0.035) in patients with elevated *Fusobacterium* relative abundance (Table 2, Fig. 1B).

### Elevated *Fusobacterium* prevalence is associated with better outcomes in mucinous CRC in the TCGA dataset

Existing evidence has linked *Fusobacterium* abundance with prognostic outcomes in CRC. To examine its precise impact with regards to mucinous tumours, we compared outcomes between patients with high and low *Fusobacterium* relative abundance, in both mucinous and non-mucinous cohorts in isolation (Fig. 2A, B). When we restricted our analysis to non-mucinous CRC patients, we found high *Fusobacterium relative* abundance did not appear to significantly impact DFS (HR 1.24, 95% CI 0.84 to 1.82, likelihood ratio test *p* = 0.29, logrank *p* = 0.28, Fig. 2B), DSS (HR 1.62, 95% CI 0.96 to 2.73, likelihood ratio test *p* = 0.08, logrank *p* = 0.07, Fig. 2B) or OS (HR 1.27, 95% CI 0.82 to 1.97, likelihood ratio test *p* = 0.29, logrank *p* = 0.29, Fig. 2B). However, univariate Cox regression models demonstrated how mucinous CRC patients with elevated *Fusobacterium* relative abundance trended to have more favourable clinical outcomes, specifically with reference to DSS (HR 0.24, 95% CI 0.05 to 1.14, likelihood ratio test *p* = 0.04, logrank *p* = 0.052, Fig. 2A).

We investigated the association between *Fusobacterium* relative abundance at higher resolution, namely at the species level, with mucinous status and clinical outcome (Sup. Fig. 1). In line with previous literature reports, we observed that *F. nucleatum* was the most abundant species (average 1.17%, 95% CI 0.00 to 10.60%), both across the whole unselected patient population and by mucinous status (mucinous: average 1.67%, 95% CI 0.00 to 11.46% vs. non-mucinous: average 1.10%, 95% CI 0.00 to 10.14%), (Sup. Fig. 1A-B). *Fusobacterium periodonticum* (average 0.15%, 95% CI 0.00% to 1.11%), *Fusobacterium necrophorum* (average 0.07%, 95% CI 0.00 to 0.22%), *Fusobacterium gonidiaformans* (average %, 95% CI 0.00 to 0.37%) and *Fusobacterium mortiferum* (average 0.04%, 95% CI 0.00 to 0.20%) were amongst the species with the highest mean relative abundance (Sup. Fig. 1A). When restricting the analysis to mucinous CRC patients, we observed an enrichment for *Fusobacterium necrophorum* (mucinous: average 0.18%, 95% CI 0.00 to 1.30% vs. non-mucinous: average 0.06%, 95% CI 0.00 to 0.21%) species (Sup. Fig. 1A).

Next, we sought to investigate the association between the relative abundance of species from the *Fusobacterium* genus with clinical outcome (OS, DSS, DFS, Sup. Fig. 1C). Univariate Cox regression models fitted on the whole unselected patients population revealed that patients with high *F. nucleatum* relative abundance have worse OS (HR 1.57, 95% CI 1.05–2.36, *p* = 0.03) and DSS (HR 1.87, 95% CI 1.16–3.03, *p* = 0.01), (grey-shaded panels, Sup. Fig. 1C). Furthermore, Cox regression models fitted with an interaction term capturing the differential impact of species abundance by mucinous status confirmed the findings at the genus taxonomic rank (light-red-shaded panels, Sup. Fig. 1C). High species relative abundance is associated with more favourable clinical outcomes in the mucinous subpopulation. In contrast, the reverse is observed in the non-mucinous subpopulation whereby high species relative abundance is associated with worse clinical outcomes.

### *Fusobacterium* abundance in rectal cancer tumour microarray validation cohort

The BRCC cohort included 15 cases (26%) of mucinous and 43 cases (74%) of non-mucinous rectal cancer, with 66% of the cohort having underwent neoadjuvant chemoradiotherapy. 14% (*n* = 2) of the mucinous cohort were MSI-high compared to 2% (*n* = 1) of the non-mucinous group. Further clinical and pathologic characteristics of the included patients are summarised in Table 3.

**Table 3:** Clinical characteristics of the patients of the BRCC cohort. RC, rectal cancer; AJCC, American Joint Committee on Cancer; Categorical data reported as *n* (%). Continuous data reported as median (IQR)

		**Mucinous RC (*N* = 15)**	**Non-Mucinous RC (*N* = 43)**	***p*-value**
**Male**		53.3% (8)	58.1% (25)	0.75
**Age**	Median (IQR)	71 (29–81)	70(42–89)	0.10
**Stage**	AJCC 1	13.3% (2)	11.6% (5)	0.17
	AJCC 2	60.0% (9)	34.8% (15)	
	AJCC 3	26.0% (4)	53.4%(23)	
	AJCC4	0.0% (0)	0.0%(0)	
**T stage**	Tis-T1-T-2	20.0% (3)	25.5% (11)	0.28
	T3	60.0% (9)	65.1% (28)	
	T4	20.0% (3)	9.3% (4)	
**N stage**	N0	73.3% (11)	48.8% (21)	0.52
	N1	6.7% (1)	41.8%(18)	
	N2	20.0% (3)	9.3%(4)	
**M stage**	M0	100% (15)	100% (43)	NA
	M1	0.0% (0)	0.0% (0)	
**Neoadjuvant CRT(*n* = 56)**^**a**^		53.3% (8)	69.8% (30)	0.32
**Adjuvant CRT (*n* = 52)**^**a**^		53.3% (8)	39.5% (17)	0.26
**MSI (*n* = 55)**^**a**^		14.3%(2)	2.4% (1)	0.09
**KRAS (*n* = 55)**^**a**^	Mutant	35.7%(5)	17.1%(7)	0.15
**BRAF (*n* = 54)**^**a**^	Mutant	7.7%(1)	2.4% (1)	0.38
**LVI (*n* = 57)**^**a**^		6.7%(1)	19%(8)	0.26
**Perineural invasion (*n* = 56)**^**a**^		14.3%(2)	11.9%(5)	0.82
**Extramural invasion (*n* = 56)**^**a**^		7.1%(1)	19.0%(8)	0.29

^a^Data not available in full cohort: n in parentheses = number with data available

**Fig. 3:**
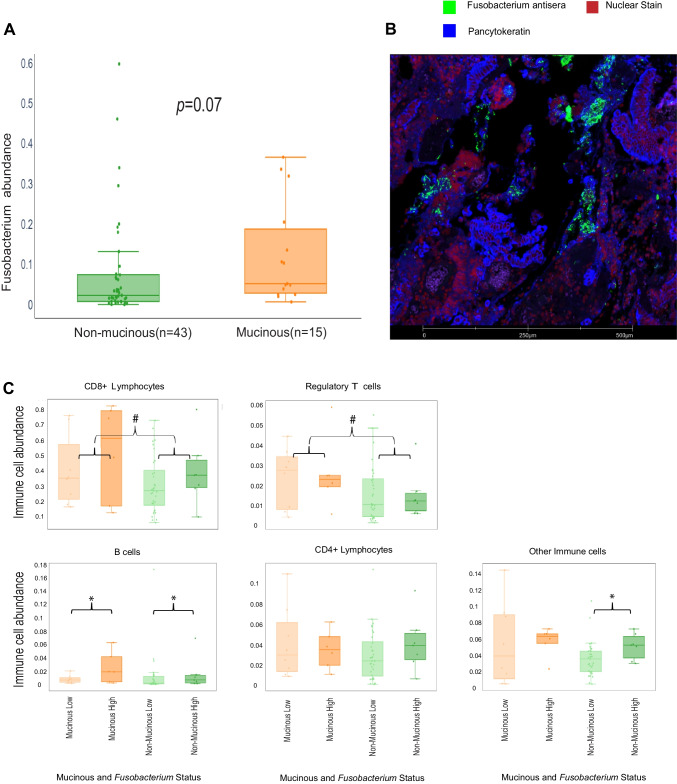
*Fusobacterium* abundance and mucinous status were found to impact immune cell expression in RC in our BRCC cohort. **A** Box and whisker blots depicting *Fusobacterium* abundance according to mucinous status. Statistical significance was evaluated using a Kruskal–Wallis test and the *p*-value is reported. **B** Image derived from the GeoMx platform of a mucinous core depicting pancytokeratin (Blue), *Fusobacterium* (Green) and Syto 13 (Red). **C** Box and whisker plots depicting specific immune cell counts; according to mucinous status and *Fusobacterium* relative abundance (high and low) within the BRCC RC cohort. Statistical significance was evaluated using Kruskal–Wallis tests. * indicates a statistically significant difference between *Fusobacterium* high and *Fusobacterium* low cohorts. # indicates a statistically significant difference between mucinous and non-mucinous cohorts

*Fusobacterium* abundance was quantified at a patient level and compared between patients with mucinous and non-mucinous RC. We again observed a trend whereby, *Fusobacterium* was more abundant in mucinous as opposed to non-mucinous RC; however, this trend fell short of statistical significance (*p* = 0.070) (Fig. 3A).

### Mucinous status and elevated *Fusobacterium* abundance are associated with increased proportions of immune cells in our rectal cancer tumour microarray validation cohort

Next, we looked to determine an association between mucinous status and *Fusobacterium* abundance with immune cell populations in our BRCC rectal cancer cohort. Mucinous rectal tumours were associated with significantly greater CD8 + lymphocyte (*p* = 0.022), regulatory T-cell (*p* = 0.047), and B-cell (*p* = 0.025) counts (Table 4, Fig. 3C).

**Table 4:** Immune cell expression in the BRCC cohort according to mucinous status and *fusobacterium* relative abundance

Cell type	Level	*p*-value
**Epithelium/stroma**	Mucinous status(mucinous) *Fusobacterium* (high) Mucinous status(mucinous): *Fusobacterium*(high)	0.914 0.384 0.491
**CD4 + lymphocytes**	Mucinous status(mucinous) *Fusobacterium* (high) Mucinous status(mucinous): *Fusobacterium*(high)	0.373 0.202 0.950
**CD8 + lymphocytes**	Mucinous status(mucinous) *Fusobacterium* (high) Mucinous status(mucinous): *Fusobacterium* (high)	**0.022** 0.097 0.491
**T regs**	Mucinous status(mucinous) *Fusobacterium* (high) Mucinous status(mucinous): *Fusobacterium* (high)	**0.047** 0.454 0.754
**B cells**	Mucinous status(mucinous) *Fusobacterium* (high) Mucinous status(mucinous): *Fusobacterium* (high)	**0.025** **0.029** 0.228
**Other immune cells**	Mucinous status(mucinous) *Fusobacterium* (high) Mucinous status(mucinous): *Fusobacterium* (high)	0.103 **0.006** 0.662

Tumours with high *Fusobacterium* abundance were found to be associated with a significantly greater proportion of B cells (*p* = 0.031) (Table 4, Fig. 3C).

## Discussion

The previously determined association between high *Fusobacterium* relative abundance and MSI status in CRC, was not found to extend to mucinous CRC in our analysis [13]. Both mucinous status and high *Fusobacterium* relative abundance were independently found to influence immune cell composition in both the TCGA CRC and BRCC RC cohorts. However, some differences existed amongst the cell types affected across our two datasets. High *Fusobacterium* relative abundance tended to be associated with improved outcomes in mucinous CRC, suggesting *Fusobacterium* may have a protective function in this specific histological subtype of colorectal adenocarcinoma.

Increased *Fusobacterium* abundance within tumour tissue has not previously been associated with positive outcomes in CRC [16]. However, findings from our group’s recent publication suggest the prognostic impact of *Fusobacterium* may differ significantly according to underlying tumour biology [11]*.* In this previous analysis, increased *Fusobacterium* colonisation was only associated with poor prognosis in mesenchymal-type tumours (CMS group 4) [11]. Our mucinous TCGA cohort was over-represented in CMS group 1, and had a far higher incidence of MSI compared to the non-mucinous group. We initially hypothesised that *Fusobacterium* may play a causative role in this context, inducing MSI thus resulting in improved outcomes in those patients with higher *Fusobacterium* relative abundance. However, our analysis demonstrated no significant association between *Fusobacterium* relative abundance and MSI status in mucinous CRC. Though this finding may simply be a reflection of the relatively smaller size of our mucinous cohort, it raises the question could the improved outcomes observed in patients with mucinous CRC with elevated *Fusobacterium* abundance, be due to factors beyond MSI status? Further analysis involving larger datasets is required to validate our preliminary findings.

The immune characteristics of mucinous CRC have been examined to a limited extent in the literature. Tozawa et al*.* demonstrated reduced peri-tumoural lymphocyte infiltration in mucinous CRC compared to non-mucinous CRC in a cohort of 152 patients [31]. Meanwhile, Nazemalhosseini-Mojarad et al. found no difference in the distribution of stromal CD8 + lymphocytes or tumour CD8 + lymphocytes in mucinous compared to non-mucinous CRC [32]. Our analysis of the TCGA dataset found immune cell proportions to be significantly greater within our mucinous CRC cohort compared to the non-mucinous group. In particular, CD8 + lymphocytes, regulatory T cells, and M2 macrophages were all found in significantly greater proportion in the mucinous cohort. Similarly, in our BRCC cohort, mucinous rectal cancer was associated with greater numbers of CD8 + lymphocytes and regulatory T cells compared to the non-mucinous group. These finding are of increased significance in the context of recently published clinical trial results, which demonstrated very encouraging outcomes for patients with MSI-high locally advanced RC, treated with immunotherapy in the neoadjuvant setting [33]. Our findings that mucinous tumours are highly immunogenic, offers hope that immunotherapy may have an important future role to play in the management of this cohort, known to demonstrate resistance to traditional adjuvant chemotherapy agents [4, 5].

Existing evidence from pre-clinical studies has linked *F. nucleatum* with recruitment of myeloid derived suppressor cells [7] and inhibition of Natural Killer cell activity [34] in CRC. Immune cell proportions varied considerably in our analysis according to the degree of tumour *Fusobacterium* abundance. Within our TCGA cohort, high *Fusobacterium* relative abundance was associated with significantly increased proportions of CD4 + lymphocytes and M1 macrophages, whilst M2 macrophages were significantly under-represented in this group. The association with macrophages in TCGA dataset could not be assessed in our BRCC cohort. M1 macrophages play an integral role in the anti-tumour immune response, via identification and direct cytotoxic effects against tumour cells [35]. Increased CD4 + lymphocyte infiltration has also been found to be associated with improved survival in mismatch repair proficient colorectal tumours [36]. Findings from a previous meta-analysis demonstrated an association between M1 macrophage and M2 macrophage infiltration and mucinous CRC, this corresponds with our analysis where M2 macrophages were over-represented in mucinous CRC [37]. High-density M2 macrophage infiltration is associated with poor survival in solid-organ tumours [37]. These cells have been implicated in tumour migration, invasion, and have been found to induce an attenuated anti-tumour immune response [37]. In the context of mucinous CRC, the finding that *Fusobacterium* are associated with a significant decrease in M2 macrophage infiltration is pertinent and may further explain how *Fusobacterium* influences outcomes positively in mucinous CRC.

Though our findings regarding the impact of *Fusobacterium* are important, there are a number of limitations to our study. Firstly, our findings are limited by the significantly smaller proportion of mucinous tumours as compared to non-mucinous across our cohorts. Preliminary findings pertaining to mucinous CRC will require further validation in a larger dataset. It is also important to note the differences in immune cell expression according to *Fusobacterium* abundance in our BRCC and TCGA cohorts. *Fusobacterium* was positively associated with B cell proportions in our BRCC cohort whilst there was no such association observed in the TCGA group. Similarly CD4 + lymphocytes were not found in greater proportion in tumours with high *Fusobacterium* abundance in our BRCC group.

Mucinous CRC is a molecularly distinct subtype of colorectal adenocarcinoma, which appears to have a unique relationship with *Fusobacterium*. *Fusobacterium* abundance may be associated with positive outcomes in mucinous CRC, this is likely through modulation of immune moderators.

## Supplementary Information

Below is the link to the electronic supplementary material.

Supplementary file1 (DOCX 14 KB)

Supplementary file2 (DOCX 250 KB)

## Data Availability

TCGA data is readily available via the TCGA repository at the National Cancer Institute (NCI) (www.cancer.gov/about-nci/organization/ccg/research/structural-genomics/tcga). Other data that support the findings of this study are available from the corresponding author upon request.
