# Supplementary material for: Increased Fusobacterium tumoural abundance affects immunogenicity in mucinous colorectal cancer and may be associated with improved clinical outcome
Source: J Mol Med (Berl). 2023 May 12;101(7):829–41. doi: 10.1007/s00109-023-02324-5 (PMC10300184; doi:10.1007/s00109-023-02324-5)
Supplement: Supplementary file 1 — Supplementary file1 (DOCX 14 KB) [file 109_2023_2324_MOESM1_ESM.docx]

| **Immune Cell** | **Expression Classification** |
| --- | --- |
| CD4+ Lymphocytes | (CD3+, CD4+ CD8-, FOXP3-) |
| CD8+ Lymophocytes | (CD3+, CD4+, CD8+, FOXP3-) |
| Regulatory T Cell | (CD3+, CD4+, CD8-, FOXP3+) |
| B Cell | (CD3-, CD4-, CD8-, CD20+) |
| Other Immune Cell | (CD3-, CD4+, CD8-, CD20-) |

**Supplementary Table 1.** Expression patterns utilised to classify immune cells.
