# Supplementary material for: Increased Fusobacterium tumoural abundance affects immunogenicity in mucinous colorectal cancer and may be associated with improved clinical outcome
Source: J Mol Med (Berl). 2023 May 12;101(7):829–41. doi: 10.1007/s00109-023-02324-5 (PMC10300184; doi:10.1007/s00109-023-02324-5)
Supplement: Supplementary file 2 — Supplementary file2 (DOCX 250 KB) [file 109_2023_2324_MOESM2_ESM.docx]

### Supplementary Figure 1


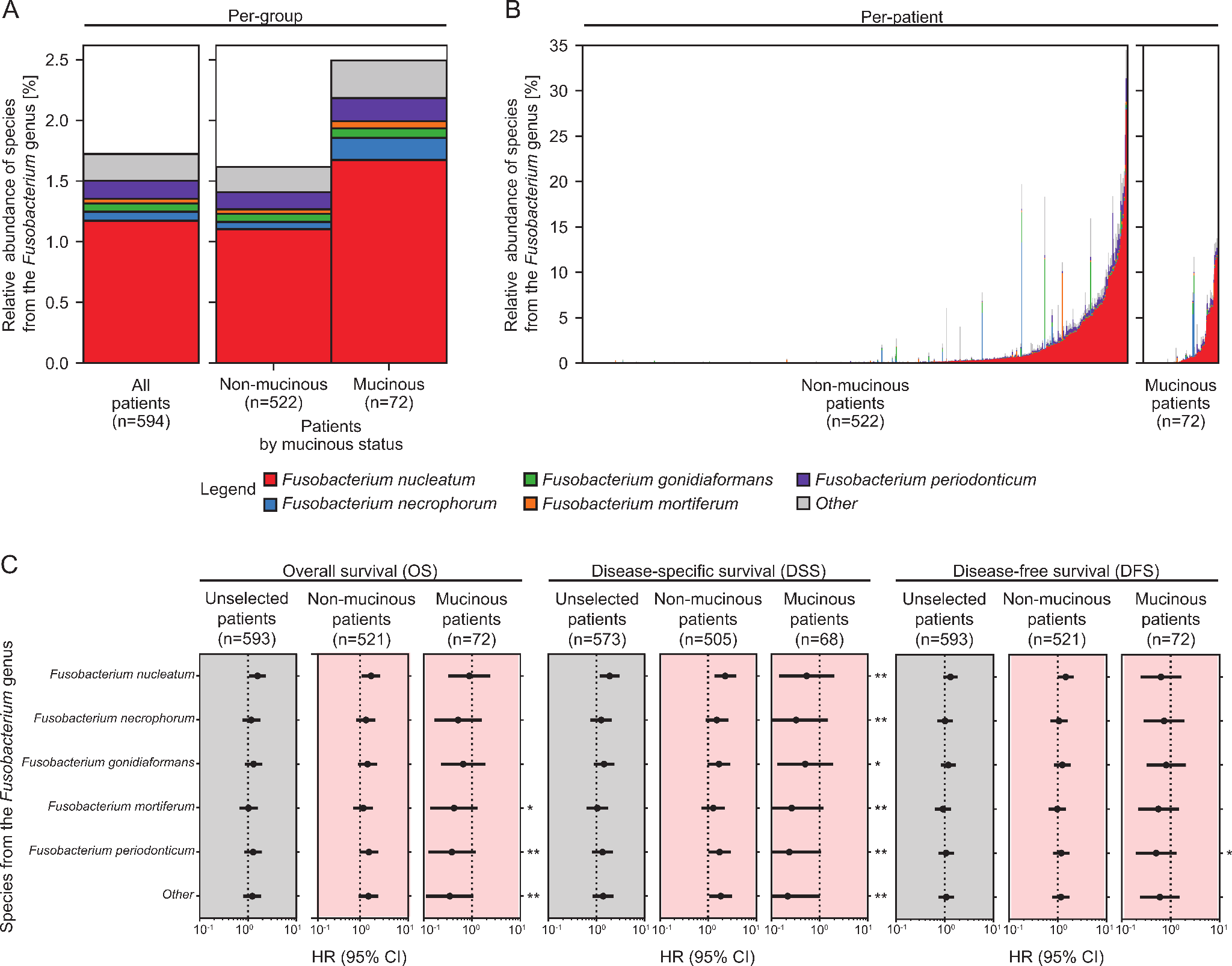


**Supplementary Figure 1.**

**A-B.** Relative abundance to total bacterial kingdom of species from the *Fusobacterium* genus shown aggregated by mean per-group, namely unselected patients population and within the mucinous and non-mucinous sub-populations, (**A**); and per-patient sorted in ascending order of *Fusobacterium* relative abundance and stratified by mucinous status (**B**).

**C.** Cox regression models were fitted on relative abundance of species from the *Fusobacterium* genus using overall- (OS), disease-specific (DSS) and disease-free (DFS) survival as clinical endpoints. For each species, patients were assigned to a low or high subgroup using the 75^th^ percentile relative abundance as threshold. The low subgroup was used as reference when reporting the hazard ratios (HRs) estimated from the Cox regression models. Univariate Cox regression models were fitted when evaluating the association between species subgroups in the whole unselected patient population (grey-shaded panels). Cox regression models with an interaction term between species subgroup and mucinous status were fitted to evaluate differential effect of species relative abundance on clinical outcome by mucinous status (light red-shaded panels). * and ** indicate statistical significance of interaction p-values below 0.1 and 0.05, respectively. Given the exploratory nature of these analyses, p-values were not adjusted for multiple comparisons.

Panels **A-C** report only top 5 most abundant species, remaining lower relative abundance species were grouped as ‘Other’.
